# Thyroid Disease Spectrum in the Emergency Department

**DOI:** 10.7759/cureus.10100

**Published:** 2020-08-28

**Authors:** Mariana Formigo, Gonçalo Castelo Branco, Magda Fernandes, Margarida Rocha, Jorge Cotter

**Affiliations:** 1 Internal Medicine, Hospital Senhora da Oliveira Guimarães, Guimarães, PRT

**Keywords:** thyroid, thyroid storm, hyperthyroidism, myxedema coma, hypothyroidism

## Abstract

We present two cases of thyroid hormone alterations revealing clinical emergencies that require early diagnosis and prompt treatment. The first patient, a 56-year-old woman, presented in the emergency room with psychomotor agitation, disorientation and headache. She was very agitated, incapable of standing still, looked very thin, feverish, tachycardic and presented no alteration at neurological examination with negative meningeal signs. Analyses revealed a severe hyperthyroidism. She initiated propylthiouracil 100 mg 8/8 h. After six months, thyroid function was normal. The second patient, a 54-year-old woman, was transferred from the Psychiatry Department due to memory and behavior changes for the past two weeks. She presented visual and auditive hallucinations and inadequate daily behavior. Analyses revealed a severe hypothyroidism. She was medicated with levothyroxine 100 ug/day. At the third month, she presented normalized thyroid function, normal thyroid ultrasound and an increased antithyroperoxidase antibody.

## Introduction

Severe alterations of the thyroid hormone are related to clinical emergencies requiring early diagnosis and prompt treatment. Although thyroid storm and myxedema coma are pathophysiologically opposed, they both present themselves as life-threatening clinical conditions associated with thyroid disruption.

Thyroid storm arises in thyrotoxic patients and is characterized by multiple organ dysfunction, usually triggered by an identified precipitant [1-4]. ​​​A high index for thyroid storm suspicion should be maintained in patients with thyrotoxicosis and any evidence of systemic decompensation [1]. Literature suggests that thyroid storm occurs in 1%-2% of hospitalized thyrotoxic patients with a mortality rate estimated at 10% [2,3].

On the other hand, patients with longstanding severe untreated hypothyroidism whose homeostatic mechanisms fail may present myxedema coma [5,6]. It is also usually triggered by a precipitating event [5]. Both hypothyroidism and myxedema coma are more common in women than in men, and myxedema coma occurs almost exclusively in patients under 60 years of age [6].

## Case presentation

Patient 1

A 56-year-old woman presented in the emergency room (ER) with psychomotor agitation, disorientation and headache. She was very agitated, incapable of standing still, looked very thin, feverish (maximum of 38.5ºC) and tachycardic (heart rate of 129-134 bpm), and neurological examination was normal. Brain CT scan showed no alterations. A lumbar puncture was done revealing 0 leucocyte and no proteinorachia. Analyses revealed a severe hyperthyroidism with T4 free of 6.85 ng/dL and thyroid-stimulating hormone (TSH) under 0.005 UI/mL (normal reference levels of T4 free are 0.76-1.46 ng/dL and TSH are 0.358-3.740 UI/mL) (Table 1).

**Table 1:** T4 free and TSH levels in patient 1 TSH, thyroid-stimulating hormone

	At admission	At discharge	First consultation (two weeks later)	Second consultation (six months later)
T4 free (reference levels: 0.76-1.46 ng/dL)	6.85	5.05	0.48	0.91
TSH (reference levels: 0.358-3.740 UI/mL	<0.05	<0.05	3.24	1.45

She had a positive benzodiazepine drug dosage, and remaining drugs and alcohol dosage were negative. She was treated with propylthiouracil 100 mg 8/8 h. On the third day, she performed an electroencephalography with no alterations. Viral serologies (HIV, toxoplasmosis, rubella, Cytomegalovirus, herpes and Epstein-Barr virus) were negative.

**Figure 1:**
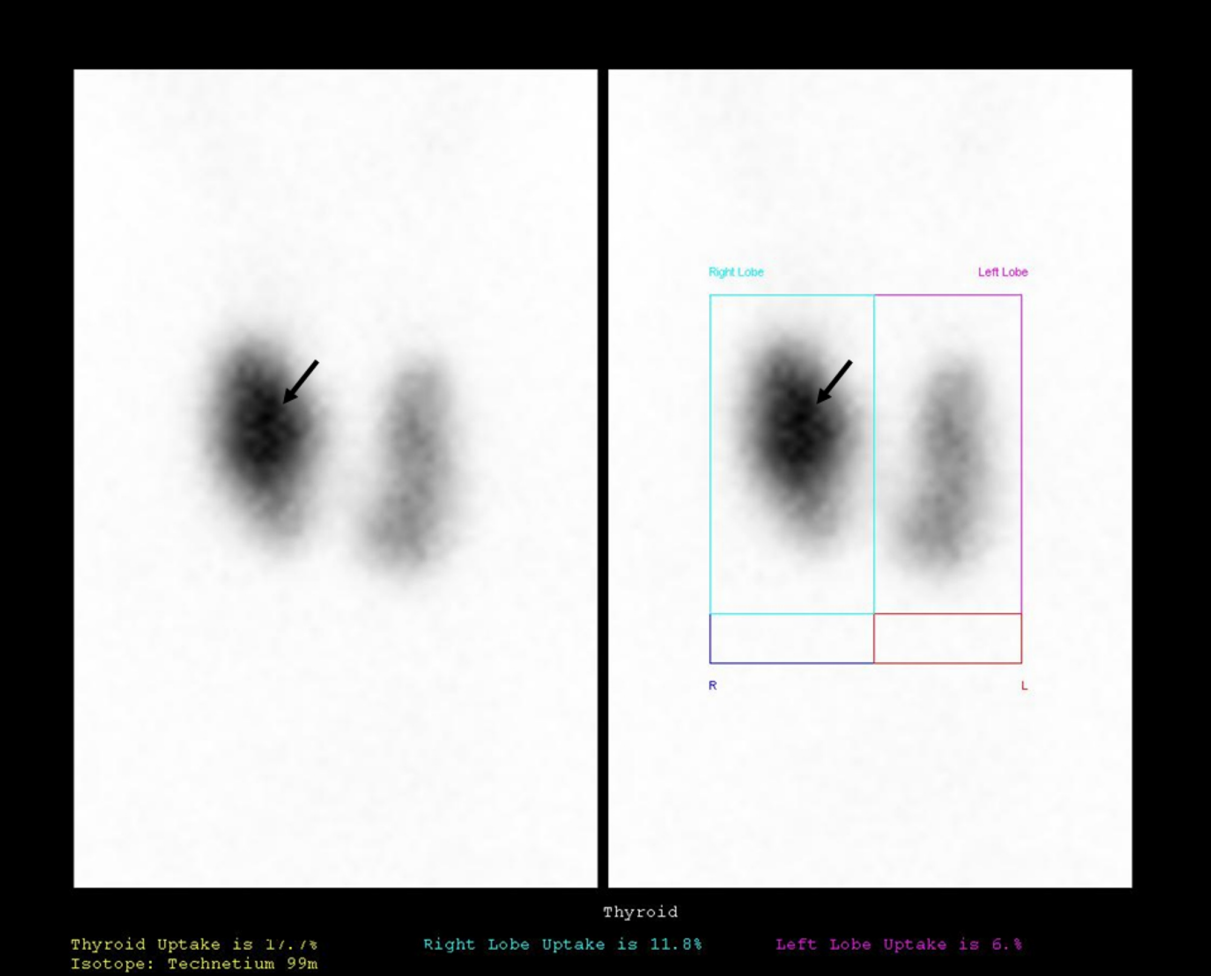
Patient 1 thyroid scintigraphy compatible with Graves’ disease with a hyperfunctioning gland with increased uptake of pertechnetate and a hyperfunctioning nodule (arrow) on the right lobe suggesting Marine-Lenhart syndrome.

On the eighth day, she performed a thyroid ultrasound revealing an enlarged thyroid with heterogeneous structure, increased glandular vascularization and bilateral follicular cysts (the largest with 9 mm on the right and 8 mm on the left) compatible with a probable condition of Graves’ disease. She had elevated TSH receptor antibodies (TRABS) of 3.94 UI/L (positive above 1.5). On the 10th day, she performed a thyroid scintigraphy compatible with Graves’ disease with a hyperfunctioning gland with increased uptake of pertechnetate and a hyperfunctioning nodule on the right lobe suggesting Marine-Lenhart syndrome (Figure 1).

She was discharged on the 11th day presenting a thyroid analysis of T4 free 5.05 ng/mL and TSH <0.005 UI/mL (Table 1). She was clinically improved, referred a minor headache but was calm and cooperative. The patient was oriented to Internal Medicine consultation two weeks later.

At the first appointment, the patient presented an iatrogenic hypothyroidism with T4 free of 0.48 ng/mL and a TSH dosage of 3.24 UI/mL. Therapeutics was adjusted and she presented a normal thyroid function after six months (Table 1).

Patient 2

A 54-year-old woman was transferred from the Psychiatry Emergency Department due to memory and behavior changes for the past two weeks. She presented somnolent, hypothermic, with visual and auditive hallucinations and inadequate daily behavior. She presented a normal neurological examination. At the ER, a cerebral computed tomography did not show any alterations. Analyses revealed a severe hypothyroidism with T4 free 0.36 ng/mL and TSH 105 UI/mL (Table 2).

**Table 2:** T4 free and TSH levels in patient 2 TSH, thyroid-stimulating hormone

	At admission	First consultation (one month later)	Second consultation (three months later)
T4 free (reference levels: 0.76-1.46 ng/dL)	0.36	1.16	0.84
TSH (reference levels: 0.358-3.740 UI/mL	105	6.2	3.4

Dosage of alcohol, drugs and HIV serology were negative. She was medicated with levothyroxine 100 ug/day and oriented for Internal Medicine consultation one month later.

At the first appointment, she maintained some psychomotor agitation and auditive hallucinations although less frequently, presented a T4 free of 1.16 ng/mL and TSH of 6.2 UI/mL (Table 2). Medication was adjusted, and she was reevaluated after three months. At the third month, she presented normalized thyroid function (T4L 0.84; TSH 3.4) (Table 2), normal thyroid ultrasound and an increased antithyroperoxidase antibody. She was clinically improved, asymptomatic, with no neurological alteration.

## Discussion

The clinical spectrum of thyroid hormone alteration ranges in severity from asymptomatic to a cataclysmic metabolic crisis needing prompt diagnosis and immediate treatment [1,2,4].

Thyroid storm pathophysiological mechanism is not yet clarified, and the diagnosis is based on clinical manifestations such as hyperpyrexia, tachycardia, arrhythmia, congestive heart failure, agitation, delirium, psychosis, stupor, coma, nausea, vomiting, diarrhea and hepatic failure [1,2,4].

Burch and Wartofsky proposed the “Burch-Wartofsky Point Scale” system for grading the severity of individual manifestations, with a point total of ≥45 consistent with thyroid storm, 25-44 points classified as impending thyroid storm and <25 points indicating that thyroid storm is unlikely [4]. According to the Point Scale for the diagnosis of Thyroid Storm by the American Endocrinology Guidelines, case 1 patient totalized 55 points (15 points for temperature of 101.3ºF, 20 points for tachycardia, 10 points for mild central nervous system manifestation with agitation and 10 points for negative precipitant history status) suggesting thyroid storm.

A multimodality treatment should be offered including therapeutics against thyroid gland blocking new hormone synthesis with propylthiouracil or methimazole, therapeutics directed against peripheral effects of thyroid hormone like glucocorticoids and beta-blockers, therapeutics directed against systemic decompensation and in severe cases monitoring in an intensive care unit [1-4].

On the other hand, myxedema coma is an uncommon and extreme manifestation of hypothyroidism. In an initial phase, these patients usually present somnolence, lethargy, fatigue, weight gain, constipation and cold intolerance; in a later phase, they present hypothermia, hypoventilation, hypotension, bradycardia, dry coarse skin, macroglossia and delayed deep-tendon reflexes [5,6].

It is a medical emergency and its early diagnosis and rapid administration of thyroid hormones and adequate supportive measures are essential for a successful outcome [5,6].

## Conclusions

Thyroid-related emergencies are rare, and their unspecific clinical manifestations may delay immediate diagnosis and worsen patients’ prognosis.

Both thyroid storm and myxedema coma are rare life-threatening complications of very common thyroid disorders. They are characterized by a multisystemic involvement and a high mortality rate. Early recognition is imperious for effective management, prompt treatment and better outcome.
